# Screening and Identification of the First Non-CRISPR/Cas9-Treated Chinese Miniature Pig With Defective Porcine Endogenous Retrovirus *pol* Genes

**DOI:** 10.3389/fimmu.2021.797608

**Published:** 2022-01-19

**Authors:** Yuyuan Ma, Junting Jia, Rui Fan, Ying Lu, Xiong Zhao, Yadi Zhong, Jierong Yang, Limin Ma, Yanlin Wang, Maomin Lv, Haiyuan Yang, Lisha Mou, Yifan Dai, Shutang Feng, Jingang Zhang

**Affiliations:** ^1^ National Medical Products Administration (NMPA) Key Laboratory for Quality Control of Blood Products, Institute of Health Service and Transfusion Medicine, Academy of Military Medical Sciences, Beijing, China; ^2^ Shenzhen Xenotransplantation Medical Engineering Research and Development Center, Shenzhen Institute of Translational Medicine, Health Science Center, The First Affiliated Hospital of Shenzhen University, Shenzhen Second People’s Hospital, Shenzhen, China; ^3^ Research and Development Department, Grand Life Science and Technology. Ltd., Beijing, China; ^4^ Department of Medical Genetics, School of Basic Medical Science, Nanjing Medical University, Nanjing, China; ^5^ Jiangsu Key Laboratory of Xenotransplantation, Nanjing Medical University, Nanjing, China

**Keywords:** porcine endogenous retrovirus, Chinese miniature pig, defective gene, inbreeding, whole-genome resequencing, full-length transcriptome sequencing

## Abstract

Pig to human xenotransplantation is considered to be a possible approach to alleviate the shortage of human allografts. Porcine endogenous retrovirus (PERV) is the most significant pathogen in xenotransplantation. We screened for pigs that consistently did not transmit human-tropic replication competent PERVs (HTRC PERVs), namely, non-transmitting pigs. Then, we conducted whole-genome resequencing and full-length transcriptome sequencing to further investigate the sequence characteristics of one non-transmitting pig. Using *in vitro* transmission assays, we found 5 (out of 105) pigs of the Chinese Wuzhishan minipig inbred line that did not transmit PERV to human cells, i.e., non-transmitting pigs. Whole-genome resequencing and full-length transcriptome sequencing of one non-transmitting pig showed that all of the *pol* genes were defective at both the genome and transcript levels. We speculate that the defective PERV *pol* genes in this pig might be attributable to the long-term inbreeding process. This discovery is promising for the development of a strain of highly homozygous and genetically stable pigs with defective PERV *pol* genes as a source animal species for xenotransplantation.

## Introduction

Pigs are considered to be the most suitable donor animals for xenotransplantation because of their anatomical and physiological similarities to humans, large litter size, short gestation period and genetic malleability (1). However, the risk of transmission of porcine microorganisms to human xenotransplant recipients is a great concern. Many exogenous microorganisms can be eliminated from donor herds by using various barrier methods and specific pathogen-free (SPF) breeding. However, porcine endogenous retroviruses (PERVs) cannot be eliminated in this way since they are integrated into the genomes of all pigs (2). PERVs can infect human cells *in vitro* under certain experimental conditions (3). Transspecies transmission has been shown for many retroviruses, including human immunodeficiency virus type 1 and 2 (HIV-1/2), human T-cell lymphotropic virus type 1 and 2 (HTLV-1/2), Koala retrovirus (KoRV) and Gibbon ape leukemia virus (GALV), and these viruses can cause more serious disease in newly infected hosts than in their natural hosts (4–8). Therefore, although transmission of PERVs has not been observed when animals (including nonhuman primates) were inoculated with PERV preparations, during preclinical xenotransplantation or in clinical transplantation to humans (9, 10), PERVs share the pathogenic potential and features common to other retroviruses and thus remain a major potential zoonotic risk in xenotransplantation.

PERVs are present in multiple copies (3 to 140 copies) in the pig genome (11). Three subtypes of PERVs were identified. PERV-A and PERV-B are present in the genome of all pigs and infect human cells as well, and PERV-C infects only pig cells and is not present in all pigs (12). In addition, recombinants of human-tropic PERV-A and ecotropic PERV-C have been detected, and one of the recombinant PERV-A/C viruses was found to be 500-fold more infectious than the prototype PERV-A (13). PERVs contain three open reading frames (ORFs), encoding *gag*, *pol* and *env*, that are located between two long terminal repeats (LTRs). The *pol* gene, encoding the reverse transcriptase, is highly conserved and is essential for PERVs’ function.

In recent years, various strategies have been proposed to reduce the risk of PERV transmission to human recipients (14), including the selection of pig strains with low expression of PERV-A and PERV-B, the selection of pigs lacking PERV-C in the genome (in order to prevent recombination between PERV-A and PERV-C), the induction of neutralizing antibodies (15–17), the generation of transgenic pigs expressing PERV-specific small interfering (si)RNA (18–21) and gene editing using either a zinc finger nuclease (22) or clustered regularly interspaced short palindromic repeats (CRISPR)/CRISPR-associated protein 9 (Cas9) technology (23, 24) to inactivate all provirus copies in the genome. Among these, CRISPR-Cas9 technology has been the most promising approach to address the PERV problem. In 2015, Yang et al. (23), using CRISPR-Cas9 technology, succeeded in inactivating 62 PERV sequences in an immortalized pig cell line. In 2017, the same group reported the successful generation of PERV-inactivated pigs by somatic cell nuclear transfer (SCNT) from primary pig fetal fibroblast cells in which PERVs have been inactivated genome-wide using the CRISPR-Cas9 system (24). This approach may minimize the risk of PERV transmission after xenotransplantation and could provide donor animals suitable for xenotransplantation.

In this study, we reported the screening and identification of the first non-CRISPR/Cas9-treated Chinese miniature pig with defective PERV *pol* genes. We screened for pigs that consistently did not transmit human-tropic replication competent PERVs (HTRC PERVs), namely, non-transmitting pigs. Then, we conducted whole-genome resequencing and full-length transcriptome sequencing to further investigate the sequence characteristics of one non-transmitting pig. The results indicated that all the PERV *pol* genes in this pig’s genome, as well as all the PERV *pol* transcripts, were defective.

## Material and Methods

### Animals

The pigs studied in this study were from a highly inbred strain of Chinese Wuzhishan minipigs (WZSPs), which was developed by the Institute of Animal Science of the Chinese Academy of Agriculture Science (CAAS) based on the inbreeding of one male and one female WZSP by full-sib mating over more than 24 generations. A total of 105 WZSPs were screened. Animal experiments were performed after receiving approval from the Beijing Experimental Animal Administration Committee (Beijing-SCXK-2015-0006) and according to the animal ethics guidelines.

### Cell Lines

The cell lines used in these studies were obtained from the American Type Culture Collection (ATCC): human embryonic kidney cell line (HEK-293 cells, ATCC CRL-1573), porcine kidney cell line (PK-15 cells, ATCC CCL-33). Cells were cultured in Dulbecco’s modified Eagle’s medium (DMEM, Gibco, Carlsbad, USA) supplemented with 5% fetal bovine serum (Zhejiang Tianhang Biological Technology Co., Ltd., Hangzhou, China). Cells were maintained by subpassaging 1 or 2 times/week as needed.

### PBMC Isolation and Activation

Blood (6 ml) was collected in ethylenediamine tetra-acetic acid tubes from each WZSP maintained by Grand Life Science and Technology Ltd., Beijing, China. Peripheral blood mononuclear cells (PBMCs) were isolated by centrifugation in lymphocyte separation medium (Tianjin HAO YANG Biological Manufacture Co., Ltd., Tianjin, China). After three washes in phosphate-buffered saline (PBS), half of the PBMCs were cryopreserved in RPMI 1640 culture medium (Gibco, Carlsbad, CA, USA) supplemented with 40% fetal bovine serum and 10% dimethyl sulfoxide. The rest of the PBMCs were mitogenically stimulated in the following medium: RPMI 1640 culture medium supplemented with 10% fetal bovine serum, 2.5 μg of phytohemagglutinin (PHA) per ml, 100 U/ml penicillin and 100 mg/ml streptomycin (Gibco, Carlsbad, CA, USA).

### *In Vitro* Coculture Assay

Briefly, HEK293 cells were plated in standard medium in a six-well plate at a density of 3×10^5^ cells per well. On the following day, approximately 6×10^5^ PHA-stimulated PBMCs isolated from the blood of WZSPs were plated onto HEK293 cells. The PBMCs were kept in contact with HEK293 cells for 4 days. After that, the culture medium and PBMCs were removed and replaced with fresh medium, and the total RNA of HEK293 cells was harvested and amplified *via* RT-PCR to detect PERV elements in the human cells. Then, the HEK293 cells cocultured with PBMCs derived from non-transmitting WZSPs were subcultured for a longer time, and HEK293 cells cocultured with PBMCs derived from a transmitting WZSP were cultured as a positive control. During this period, as HEK293 cells reached confluence, culture medium was collected for reverse transcriptase (RT) assays, and 10^6^ cells were collected for genomic DNA and RNA isolation and subsequent PCR and RT-PCR test.

### PCR and RT-PCR

Genomic DNA and total RNA were extracted from HEK293 cells with TRI Reagent (Sigma-Aldrich, Merck KGaA, Darmstadt, Germany) according to the manufacturers’ instructions. Genomic DNA was used as a template for detecting pig GGTA1 to control for potential porcine genome contamination of HEK293 cells after coculture. Three microliters of DNA was added to a reaction mixture (in a final volume of 25 μl) containing 12.5 μl Premix Taq (Takara Biomedical Technology (Beijing) Co., Ltd., Dalian, China) and 1 pmol of each GGTA1 primer. Amplification was performed on a Veriti 96-well Thermal Cycler (Applied Biosystems, California, USA) under the following conditions: 1 cycle of 94°C for 5 min; 35 cycles of 94°C for 30 s, 56°C for 50 s, and 72°C for 45 s; and a final elongation step at 72°C for 5 min. RNA was converted to cDNA with M-MuLV reverse transcriptase (New England Biolabs, Ipswich, MA, USA) according to the manufacturer’s protocols. Genomic DNA and cDNA of HEK293 cells were then tested by PCR to detect PERV elements (*gag*, *pol* and *env*) in the human cells using three primer pairs as described previously (25). The cDNA of PK15 cells was used as a positive control. All the amplified products were evaluated by 1.5% agarose gel electrophoresis with ethidium bromide staining.

### Reverse Transcriptase Activity Assay

RT activity in the culture supernatants was assayed by using an HS-Mn RT Activity Kit (Cavidi AB, Uppsala, Sweden) according to the manufacturer’s protocol.

### Whole-Genome Resequencing and Sequence Analyses

Genomic DNA was extracted from PBMCs of WZSP452 using a Wizard^®^ genomic DNA purification kit (Promega (Beijing) Biotech Co., Ltd., Beijing, China) and then submitted to iGeneTech Bioscience Co., Ltd. (Beijing, China) for whole-genome resequencing on an Illumina HiSeq 4000 system (Illumina Inc., San Diego, CA, USA). Library preparation and sequencing as well as sequence data processing were conducted as previously described (26). After quality filtering, the remaining reads were mapped to the genomic data from the *Sus scrofa* breed Wuzhishan isolate L1-53 (GenBank accession number: AJKK01000000.1) using Burrows-Wheeler Alignment (BWA) (27). Then, the sequence of full-length proviral DNA of PERV-WZSP (GenBank accession number: EF133960.1), which had been previously derived from WZSP and sequenced by our group (28), was used as the reference sequence of PERV for the following sequence analysis. The pig genomes of WZSP452, Wuzhishan isolate L1-53 (GenBank accession number: AJKK01000000.1) and Duroc isolate TJ Tabasco (GenBank accession number: AEMK00000000.2) were screened with the *pol* gene of the PERV reference sequence by using SAMtools-0.1.19 (29). Then, the complete genome of the PERV-WZSP sequence was mapped onto the WZSP452 genomes with BLAST to detect full-length PERV insertions. The characteristics of PERV protein genes integrated into the WZSP452 genome were analyzed using the DNA Star software package (30).

### Validation of Assembly *pol* Sequences in Scaffold640 and Scaffold5028 of WZSP452

The *pol* sequence in Scaffold640 contains 426 gaps (indicated by N). We designed the primers Scaffold640-polF (5’-AAGGGAAACAAAGGACTGAAGG-3’) and Scaffold640-polR (5’-GAGTTCAGGCTGTCTCCTATGC-3’) to close the gaps in Scaffold640 based on their flanking regions, and the fragment length amplified by this pair of primers is 3400 bp. Genomic DNA (6 µl) extracted from PBMCs of WZSP452 was added to a reaction mixture (in a final volume of 50 μl) containing 25 μl Q5^®^ Hot Start High-Fidelity 2× Master Mix (New England Biolabs, Ipswich, MA, USA) and 5 pmol of each Scaffold640-*pol* primer. Amplification was performed on a Veriti 96-well Thermal Cycler (Applied Biosystems, California, USA) under the following conditions: 1 cycle of 94°C for 30 s; 35 cycles of 94°C for 10 s, 57°C for 30 s, and 65°C for 100 s; and a final elongation step at 65°C for 10 min. After treatment with Premix Ex Taq (Takara Biomedical Technology (Beijing) Co., Ltd., Dalian, China), the resultant deoxyadenosine triphosphate (dA)-tailed PCR products were characterized by 1% agarose gel electrophoresis and extracted using a TIANgel Midi Purification Kit (TIANGEN Biotech (Beijing) Co., Ltd., Beijing, China). Purified products were ligated into the pMD18-T vector (Takara Biomedical Technology (Beijing) Co., Ltd., Dalian, China) according to the manufacturer’s instructions and transformed into *E. coli* DH5α competent cells (Takara Biomedical Technology (Beijing) Co., Ltd., Dalian, China). Plasmids were purified using the PureYield™ Plasmid Miniprep System (Promega (Beijing) Biotech Co., Ltd., Beijing, China). The insert size was confirmed by restriction digestion and analysis (*Sal*I and *Xba*I) (New England Biolabs, Ipswich, MA, USA). The plasmids containing inserts of the correct size were submitted to Beijing Biomed company (Beijing, China) for Sanger sequencing. Then, the sequences were aligned with the sequence of Scaffold640 using the DNA Star software package (30).

With regard to Scaffold5028, based on flanking regions of the premature termination codon of the PERV-*pol* gene, PCR primers Scaffold5028-*pol*F (5’-ACTTGGGAGTGGGACGGGTAAC-3’) and Scaffold5028-*pol*R (5’-AATCCATCCCTGCGGTTTCTAC-3’) were designed to amplify a 276 bp product. Genomic DNA (2 µl) extracted from PBMCs of WZSP452 was added to a reaction mixture (in a final volume of 50 μl) containing 25 μl Q5^®^ Hot Start High-Fidelity 2× Master Mix (New England Biolabs, Ipswich, MA, USA) and 2 pmol of each Scaffold5028-*pol* primer. Amplification was performed on a Veriti 96-well Thermal Cycler (Applied Biosystems, California, USA) under the following conditions: 1 cycle of 98°C for 30 s; 35 cycles of 98°C for 10 s, 64°C for 30 s, and 72°C for 100 s; and a final elongation step at 72°C for 2 min. The resultant PCR products were submitted to Allwegene Tech. (Beijing, China) for high-throughput next-generation sequencing on the Illumina MiSeq platform (PE300).

### Full-Length Transcriptome Sequencing

Transcriptome sequencing was performed by using single-molecule real-time (SMRT) sequencing, developed by Pacific Biosciences (PacBio). Briefly, total RNA was extracted from PBMCs derived from WZSP452 using TRI Reagent (Sigma-Aldrich, Merck KGaA, Darmstadt, Germany) according to the manufacturers’ protocol. Then, according to the Isoform Sequencing protocol (Iso-Seq), Iso-Seq libraries were prepared using the SMARTer PCR cDNA Synthesis Kit (Clontech, Mountain View, CA, USA) and the BluePippin™ Size Selection System (Sage Science, Beverly, MA, USA) as described by Pacific Biosciences (Part Number 100-092-800-03). Sequencing was carried out on the Pacific Bioscience RS II platform (Pacific Biosciences, Menlo Park, CA, USA).

The raw data obtained by sequencing were processed using SMRTlink 5.0 software. Circular consensus sequences (CCSs) were generated from subread BAM files and then classified as full-length or non-full-length reads using ‘pbclassify.py’. Reads without poly(A) tail signals and short reads (minimum sequence length = 200) were discarded. The full-length reads were then fed into the isoform-level clustering, namely, iterative clustering for error correction (ICE), followed by polishing using the Arrow function. Additional nucleotide errors in consensus reads were corrected using the Illumina RNA-seq data with the software LoRDEC (31). The consensus reads were aligned with GMAP (32) to the pig genome sequence and to the genome of the PERV-WZSP isolate (GenBank accession number: EF133960.1).

## Results

### *In Vitro* Transmission Assays for Screening WZSPs With Non-Transmitting PERV

We conducted *in vitro* transmission assays to screen for WZSPs that did not transmit PERVs from pig to human cells. After coculture with PHA-stimulated PBMCs derived from each WZSP for 4 days, total RNA was extracted from the HEK293 cells and amplified *via* RT-PCR to detect the expression of PERV *gag*, *pol*, and *env* genes in the human cells. Overall, we screened 105 WZSPs, and 17 WZSPs tested negative in the assay.

**Figure 1:**
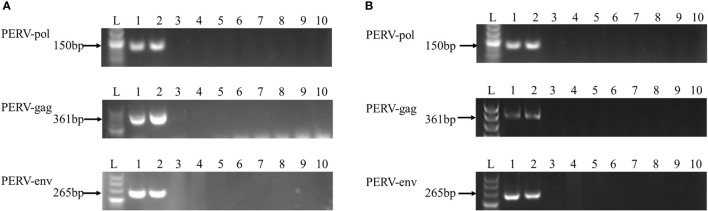
PCR **(A)** and RT-PCR **(B)** analysis of PERV elements. Agarose gel electrophoresis was performed with PERV-pol, PERV-gag, and PERV-env PCR and RT-PCR products obtained from DNA **(A)** and RNA **(B)** prepared from HEK293 cells cocultured with the PBMC derived from the following WZSPs: lane 2, WZSP153 (a transmitting WZSP, as a positive control); lane 3-9, WZSP169, WZSP456, WZSP444, WZSP129, WZSP1213, WZSP505, WZSP452. Lane 1, positive control (PK15 cells); lane 10, no template control (NTC). The sizes of the products obtained are indicated to the left.

Subsequently, the HEK293 cells cocultured with PBMCs derived from 8 out of 17 abovementioned WZSPs were maintained for a longer time, as were those derived from the positive control WZSP153 (a pig with transmitting PERV). During this period, genomic DNA as well as total RNA of the HEK293 cells were collected at each cell passage, followed by PCR and RT-PCR analysis for PERV detection. PERV infection of HEK293 cells was also determined by the measurement of reverse transcriptase (RT) activity in the HEK293 cell culture supernatant. Finally, 5 out of 8 WZSPs were confirmed to be negative for all these assays (**Figure 1** and **Table 1**). These 5 WZSPs could be considered to have non-transmitting PERV, that is, there was no *in vitro* transmission of PERV from pig to human cells.

**Table 1:** Reverse transcriptase (RT) activity in the supernatant of HEK293 cell cocultured with PBMC derived from WZSPs.

Pig No.	RT activity (μU/ml) in the supernatant of HEK293 cells on following days passaged																						
	Days post coculture with PBMC derived from WZSPs																						
	2	4	7	9	10	14	16	18	19	21	24	27	28	33	35	40	42	46	47	49	51	54	61
169	–		–			–				–			–		–		–			1450.74			
456				–					–			–		–		–			–			–	–
444				–					–			–		–		–			–			–	–
129	–		–					–		–			–		–		–				647.78		
1213				–					–			–		–		–			–			–	–
505	–		–			–				–			–		–		–	–					
452	–		–			–				–			–		–		–				–		
1209	–	–		–	–	–	–			421.18	445.81												
**153**				–					–			658.65		475.96									

Shown are data of RT activity in the supernatant of HEK293 cell cocultured with PBMC derived from WZSPs for approximately 24-61 days. Blank ﬁelds indicate that the RT activity was not tested on that day. “–” indicates that the RT activity was negative. 153 is a transmitting WZSP, which is used as the positive control.

### Whole-Genome Resequencing of WZSPs With Non-Transmitting PERV

We conducted whole-genome resequencing on one of these non-transmitting pigs (i.e., WZSP452) to further investigate the sequence characteristics. We compared the quality control gene fragment with the reference genome (Wuzhishan Inbred Pig GenBank: AJKK01000000.1). The average depth was 13.55×, and these mapped reads covered 95.03% of the swine reference assembly. The genomes of WZSP452, Wuzhishan isolate L1-53 (GenBank No.: AJKK01000000.1) and Duroc isolate TJ Tabasco (GenBank No.: AEMK00000000.2) were searched for the *pol* gene of PERV-WZSP. In total, 95 *pol* gene fragments were detected in WZSP452, with 1 fragment longer than 3000 bp, 4 fragments between 1000 and 3000 bp, and the remaining fragments all shorter than 1000 bp. None of these *pol* gene fragments were intact. We also found a similar scenario in Wuzhishan isolate L1-53. In Duroc isolate TJ Tabasco, 49 *pol* gene fragments were detected, with 18 fragments longer than 3000 bp, 9 fragments between 1000 and 3000 bp and 22 fragments shorter than 1000 bp. Among them, 8 *pol* gene fragments were intact (**Table 2**).

**Table 2:** Copy number of PERV-*pol* gene in pig genomes.

Host	Copy number of PERV-*pol* gene fragments					
	Total copies	Fragments >3000 bp in length	Fragments between 1000 and 3000 bp in length	Fragments <1000 bp in length	Defective genes	Intact genes
Duroc isolate TJ Tabasco	49	18	9	22	41	8
Wuzhishan isolate L1-53	95	1	4	90	95	0
WZSP452	95	1	4	90	95	0

**Figure 2:**
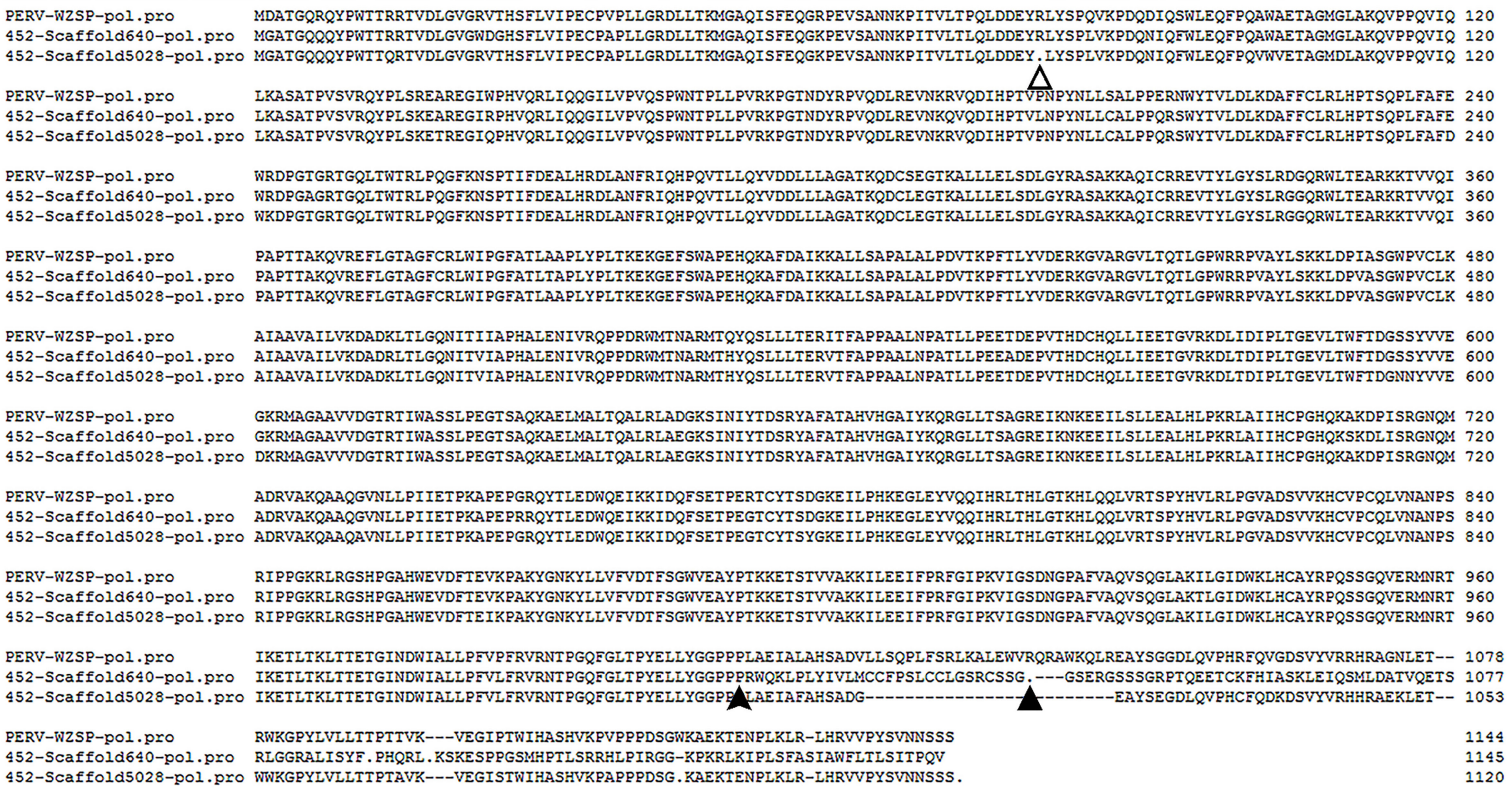
Alignment of the deduced amino acid sequences of the PERV *pol* from Scaffold640 and Scaffold5028 with that of PERV-WZSP isolate (GenBank accession number: EF133960.1). The filled arrowhead indicates the position of a two base-pair (CC) addition in the sequence of Scaffold640, which results in a frameshift mutation and premature termination at peptide position 1038 (indicated by the filled triangle). The unfilled triangle indicates an A>T mutation in the sequence of Scaffold5028, which leads to a premature stop codon and resulted in a protein sequence truncated to 78 amino acids.

We next searched the genome of WZSP452 for the nucleotide sequences of PERV-WZSP-encoding proteins (*gag*, *pol, env*) with the following parameters: e-value cutoff 1e-10, ≥80% sequence identity, and ≥80% length coverage. A total of 8 PERV-derived protein-coding genes were identified, including 1 *gag* gene, 2 *pol* genes and 5 *env* genes. Only 1 copy of the complete sequence of PERV, integrated into Scaffold5028, was detected in the genome of WZSP452 (**Table 3**), consistent with the results when screened with the complete genome of PERV reference sequence. The 2 assembly *pol* sequences were further validated by PCR and subsequent sequencing analysis. A sequence gap of 690 bp within the *pol* gene region of Scaffold640 was filled with Sanger sequence data generated with primers that flanked the gap. The complete *pol* gene of Scaffold640 was 3437 bp in length. Compared with the *pol* sequence of the PERV-WZSP genome, a 2-bp (CC) insertion after nt 3026 of Scaffold640-*pol* resulted in a frameshift mutation and premature termination at peptide position 1038. The *pol* gene of Scaffold5028 was 3360 bp in length and carried a 235A>T mutation, confirmed by next-generation sequencing, that led to a premature stop codon and resulted in a protein sequence truncated to 78 amino acids compared to the normal protein with 1144 amino acids (**Figure 2**).

**Table 3:** Location of PERV genome integrated into the WZSP452 genome.

WZSP452Scaffolds	Strand	Length	Start	End	Viral protein genes included
Scaffold12	–	2776	321642	318867	env
Scaffold42	–	2153	299876	297724	env
Scaffold226	+	3431	443655	447103	env
Scaffold640	+	6955	1381011	1387965	pol, env
Scaffold5028	+	8875	23882	32756	gag, pol, env

These results indicated that all of the *pol* genes in the WZSP452 pig genome were defective. Furthermore, the *gag* and *env* genes of Scaffold5028 were defective as well, with lengths of 1574 and 1964 bp and premature termination at peptide positions 11 and 137, respectively.

### Full-Length Transcriptome Analysis of WZSPs With Non-Transmitting PERV

WZSP452 pig transcriptomes were further sequenced using the PacBio platform to analyze the expression profile of PERV-*pol*. PacBio sequencing yielded a total of 29,8126 polished consensus reads. The mean length of all resulting transcripts was 2751 bp, ranging from 192 to 17,500 bp.

**Figure 3:**
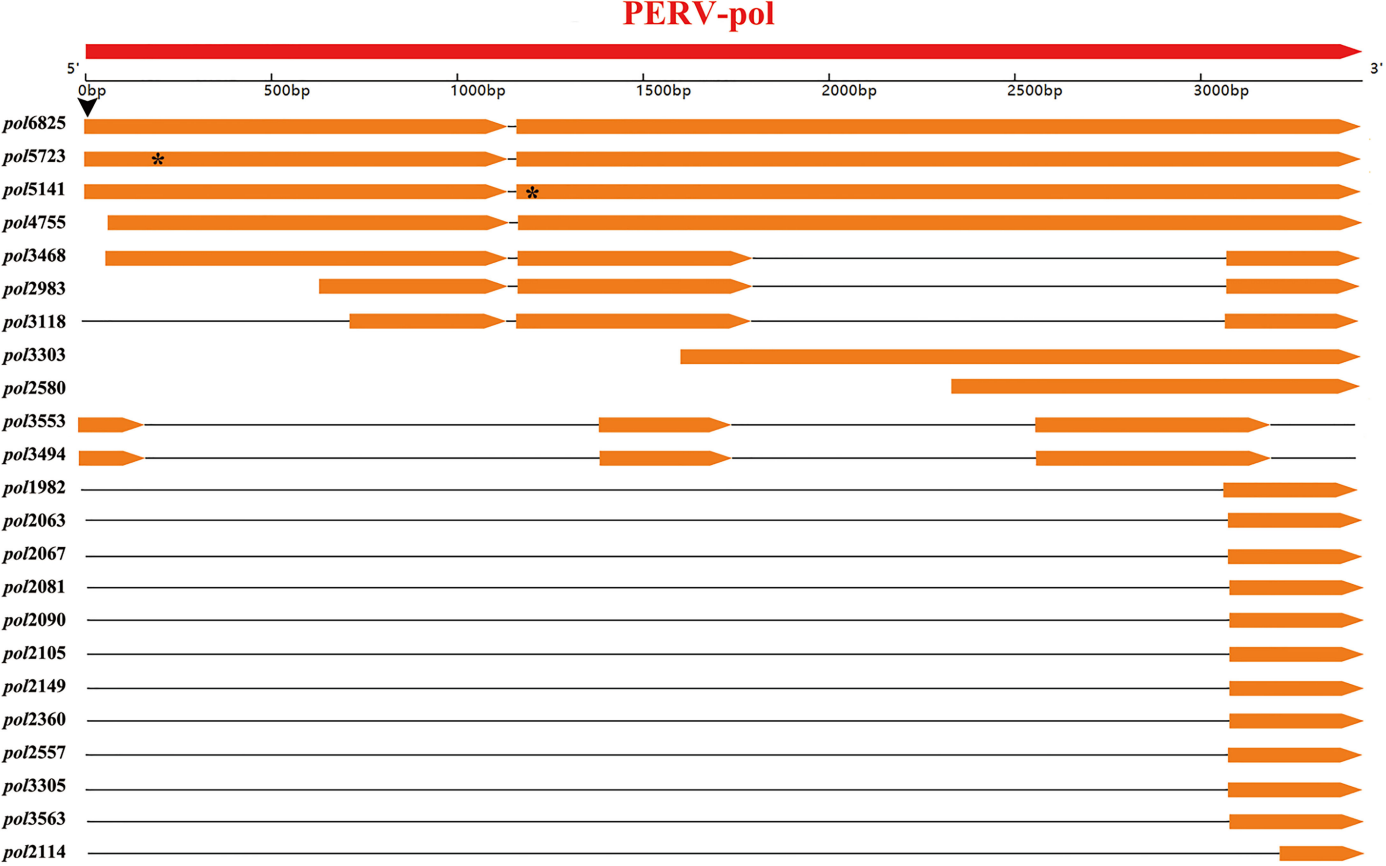
Location of the detected 23 transcripts of WZSP425 on the PERV-WZSP *pol* gene. Arrow-rectangles in red indicated the ORF of PERV-WZSP *pol* gene, arrow-rectangles in orange indicated the detected 23 transcripts of WZSP452. Horizontal black lines represented deletions or introns of the gene. The black arrowhead indicated the position of a single base mutation (A>G), which results in the loss of start codon ATG of *pol*6825. The “*” symbol marked the positions of the premature stop codons in the *pol* gene from the *pol*5723 and *pol*5141.

These transcripts were aligned to the genome of the PERV-WZSP isolate by BLAT search and screened against PERV protein sequences by BLASTX with identity of ≥60% and length of ≥60 bp set as criteria. Then, the detected transcripts were aligned with the *pol* sequence of PERV-WZSP. Consequently, 23 transcripts that harbored the PERV-*pol* sequence were detected and named after their length (**Figure 3**). Of the 23 transcripts detected, 3 could be mapped to the full-length sequence of the *pol* gene. Sequence analysis further indicated that *pol*6825 revealed an A>G transversion at the first base of the start codon, leading to the loss of the start codon ATG (methionine) of the *pol* gene. In *pol*5723, we found a single base deletion at 209 nt causing a frameshift and a consequent premature stop codon at peptide position 70. In *pol*5141, the 23 nt deletion after the 1137 nt position led to an altered amino acid sequence and introduced a premature stop codon at peptide position 400.

The remaining 20 transcripts harbored incomplete *pol* gene sequences, and they were located as follows: two transcripts mapped from nts 60 to 3435 on the PERV-*pol* gene, and one of them had a deletion from nts 1802 to 3073; two transcripts mapped from nts 642 to 3434 and from nts 721 to 3435, respectively, and both of them had a deletion of nts 1802 to 3073; one transcript mapped from nts 1605 to 3435; one transcript mapped from nts 2336 to 3435; two transcripts mapped from nts 1 to 3208 with 2 deletions (nt 194-1414 and nt 1762-2561); eleven transcripts mapped from nts 3074 to 3435; and one transcript mapped from nts 3094 to 3435 with a deletion of nts 3156 to 3219.

These results indicated that all *pol* transcripts in the WZSP452 pig were unlikely to produce functional POL proteins.

## Discussion

To address the potential risk of PERV transmission in pig-to-human xenotransplantation, scientists have tried multiple approaches for years. During recent years, the successful generation of live piglets in which PERV genes were inactivated using CRISPR-Cas9 technology has attracted considerable attention. Undoubtedly, the generated PERV-inactivated pigs could be considered a remarkable breakthrough, as these pigs could serve as organ donors for future clinical xenotransplantation. This aside, using conventional animals (non-CRISPR/Cas9-treated animals) in well-controlled trials is believed to be feasible and has been performed in the past (10). Apart from pigs with the absence of PERV-C and low copy number and low expression of PERV-A and PERV-B, pigs that do not transmit human-tropic replication-competent PERVs (HTRC PERVs), namely, “non-transmitting pigs”, could be an option.

In 2002, Oldmixon BA (33) investigated the PERV transmission characteristics of a unique inbred herd of miniature swine, with the result that inbred miniature swine that consistently do not transmit HTRC PERVs can be identified, and two of these lines of miniature swine (SLA d/d and SLA g/g) showed low incidences of transmission. In 2004, Wood JC (34) identified a group of miniature swine that do not carry PERV that infects either human or pig cells; these animals are referred to having a PERV-null transmission phenotype. Mainly using the coculture infectivity test with human HEK293 or porcine ST-IOWA target cell lines, Garkavenko O (35) established that Auckland Islands pigs have an extremely low risk of transmitting PERV infection and can therefore be qualified as “null” pigs. Islet cells from these well-characterized Auckland island pigs were used for two clinical trials that have been performed to treat diabetes in humans in New Zealand and Argentina (36, 37). In all cases, a positive medical effect was observed, and neither PERVs nor other porcine viruses under investigation were transmitted to the transplant recipients.

In the present study, we screened Chinese WZSP inbred pigs and found that 5 pigs consistently did not transmit HTRC PERVs in the *in vitro* coculture assays recommended by the U.S. Food and Drug Administration (FDA) (38). We further demonstrated that WZSP (WZSP452) was characterized by defective PERV *pol* genes at both the genomic DNA and transcriptome levels. To the best of our knowledge, this is the first report of a non-CRISPR/Cas9-treated Chinese miniature pig with defective porcine endogenous retrovirus *pol* genes. The WZSP inbred line in this study was developed based on the inbreeding of one male and one female WZSP by full-sib mating over more than 27 generations (39). These inbred WZSPs are characterized by a high level of homozygosity and genetic stability (40, 41). It has been demonstrated that genomic regions with extremely low rates of heterozygosis (<0.001%) account for 60% of the WZSP inbred line genome (40), providing a theoretical basis for massive gene loss during inbreeding. The defects in the PERV *pol* genes might be attributed to mutations, deletions, and insertions of proviral gene fragments accumulated during the long-term inbreeding process.

Further identification of more WZSPs with defective PERV *pol* genes and inbreeding might lead to the generation of a highly homozygous and genetically stable pig strain with defective PERV *pol* genes. This is a cost-effective strategy compared to gene editing combined with somatic cell nuclear transfer and, to some extent, would be safer. This strain could be a promising source of tissues and organs for xenotransplantation.

However, since the defective sites in different pigs could be different, we could not completely exclude the possibility that recombination and complementation of intact ORFs from two defective proviral genomes could lead to infectious PERVs. Similarly, two defective RNAs can theoretically be packaged into a single viral particle and give rise to infectious virus by recombination and complementation, though the likelihood of such an event is considered to be low (42, 43). Therefore, further identification and monitoring of the PERV transmission characteristics of this pig strain are necessary.

In summary, the present study screened and identified the first non-CRISPR/Cas9-treated Chinese miniature pig with defective PERV pol genes. The development of a strain of highly homozygous and genetically stable pigs with defective PERV pol genes as source animal species is promising for xenotransplantation.

## Data Availability Statement

The whole genome sequence data and transcriptome data generated in this study have been deposited in NCBI Sequence Read Archive, under the accession number SRR6832872 and SRR10914223, respectively. The Sanger sequencing data of the pol sequence in Scaffold640 of WZSP452 have also been deposited at GenBank under the accession number MH198419.

## Ethics Statement

The animal study was reviewed and approved by Beijing Experimental Animal Administration Committee.

## Author Contributions

YM, JJ, RF, and YL contributed equally. YM, JJ, RF, and YL performed most of the experiments and analyzed the data. YM and JJ wrote the paper. XZ, YZ, JY, and LMa collected samples and performed the experiments. YW, ML, and HY critically revised the manuscript. LMo and YD helped with result interpretation. SF and JZ conceived the project. All authors reviewed the draft before submission. All authors contributed to the article and approved the submitted version.

## Funding

This work was supported by National Natural Science Foundation of China (32070538, 31502060 and 81874144) and National Key R&D Program of China (2017YFC1103701 and 2017YFC1103704).

## Conflict of Interest

Authors SF and JY are employed by Grand Life Science and Technology, Ltd.

The remaining authors declare that the research was conducted in the absence of any commercial or financial relationships that could be construed as a potential conflict of interest.

## Publisher’s Note

All claims expressed in this article are solely those of the authors and do not necessarily represent those of their affiliated organizations, or those of the publisher, the editors and the reviewers. Any product that may be evaluated in this article, or claim that may be made by its manufacturer, is not guaranteed or endorsed by the publisher.
